# A Personal Health App and Wearable Co-Design Framework for Rare and Complex Diseases: User-Centered, Collaborative Co-Design Study

**DOI:** 10.2196/85425

**Published:** 2026-07-20

**Authors:** Sarah Margaret Goodday, Emma Karlin, Madeleine Sorensen, Robin Yang, Paul Gordon, Caresse Opoku, Daniel Vuong, Jenee Wilson, Diane McKenzie, Jules Piccotti, Massimiliano Tavanti, Megan Fitzgerald, Yochai Re'em, Hannah Wei, Megan Golden, Daniel Morgan, Michele Manion, Ricardo Mosquera, Tricha Shivas, Elise Hoover, Allison Peck, Nathan Peck, Zollie Yavarow, Heidi Bjornson-Pennell, Andra Stratton, Samali Anova Sahoo, Douglas Arbittier, Elizabeth Arbittier, Renee Goff, Donna Harvin-Graham, Nancy Howard, Camille Knudsen, Mary Oldham, Stephen Friend

**Affiliations:** 1 Department of Psychiatry University of Oxford Oxford United Kingdom; 2 4YouandMe New York, NY United States; 3 AgileOps Siena Italy; 4 Patient-Led Research Collaborative Calabasis, DC United States; 5 Mission: Cure New York, NY United States; 6 Primary Ciliary Dyskinesia (PCD) Foundation Rochester, NY United States; 7 Department of Pediatrics The University of Texas Health Science Center at Houston Houston, TX United States; 8 Foundation for Sarcoidosis Research Chicago, IL United States; 9 Cure VCP Disease Inc. Warner Robins, GA United States; 10 Biohub San Francisco, CA United States; 11 University of Pennsylvania Philadelphia, PA United States

**Keywords:** co-design, digital health, l ong COVID-19, mobile health, pancreatitis, primary ciliary dyskinesia, PCD, sarcoidosis, user-centered, valosin-containing protein, VCP disease, wearables

## Abstract

**Background:**

End user co-design in the personal digital health technology space is underdeveloped. Clinical uptake of personal digital health technologies has been poor, highlighting a need to cocreate solutions with end users.

**Objective:**

The study aimed to describe an “end user” co-design framework in the development of 5 prototype personal health apps for patients with different rare or complex diseases.

**Methods:**

A patient-led, user-centered, collaborative personal health app plus wearable plug-in co-design methodology was developed. Five prototype apps were developed for end users with long COVID-19, pancreatitis, primary ciliary dyskinesia, sarcoidosis, and valosin-containing protein disease by a multidisciplinary partnership including patients, app design and development experts, user experience experts, clinicians, and patient-driven organizations. Phase 1 involved a 6-month co-design process with 5 modules involving patient-driven organizations that included the codevelopment of specifications through group workshops and independent exercises that defined the goals, content, features, and user experience of each app. Phase 2 involved app build-out, internal alpha testing, and beta study preparations. Phase 3 involved a usability beta testing study in which end users used the app and associated wearable/smart devices (Oura ring, Lumia ear device, Empatica EmbracePlus, and MIR Spirobank Spirometer) for up to 5 months. Participant feedback was documented continuously and systematically, centering on the following themes: functionality, usability, harms, benefits, self-explorations, and beta testing study details related to retention and adherence.

**Results:**

While unique app goals were codeveloped by each disease group, a central goal across groups was to develop a personal health app enabling users to track subjective, self-reported symptoms, objective measures of health, and unique modifiers of symptoms. A total of 239 end user participants participated in the beta testing pilot study. Enrollment and retention rates were high, ranging from 94% to 100% and 92.2% to 100%, respectively. All active participants gave some form of feedback: there were 257 unique participant suggestions of how to specifically modify or improve the study app experience. Participant feedback themes commonly centered around customization to reduce daily burden and improve personal tailoring of the app. Participants’ desires surrounding symptom displays were heterogeneous.

**Conclusions:**

Personal health app co-design is rooted in a complex digital landscape that requires a significant amount of up-front effort and time. However, the up-front investment of time can result in rich and diverse end user feedback that could save time in the app development trajectory to implementation. This paper provides a co-design framework and the building blocks of 5 prototype personal health apps with publicly available open-source code on GitHub. These prototypes could be leveraged for improving understanding of, communicating symptoms of, and providing n-of-1 suggestions for rare or complex diseases, providing benefit to patient communities and individual patients.

## Introduction

Rare and novel conditions can present unique challenges for researchers, clinicians, and patients due to their low prevalence, phenotypic heterogeneity, and lack of natural history data. For rare and novel conditions, there is limited understanding of factors that trigger or modify symptom and disease progression. There is a need for patients to be better equipped with tools to follow their symptoms, along with insights about what improves and worsens their symptoms; for clinicians to be equipped with more accurate knowledge to help guide their patients; and for patient communities to better understand natural histories of their disease to guide research and clinical activities.

Personal digital health technologies (P-DHTs [1]) such as smartphone apps and wearable devices harness the potential for real-world, high-resolution, and longitudinal health monitoring at the individual context [2]. Consumer-grade wearable and smart devices are capable of tracking semicontinuous biometric measures (eg, heart rate, heart rate variability, body temperature, blood pressure, oxygen saturation, and respiratory function), behavior (eg, activity, relative location, and sleep duration and quality), and social activity (eg, social media and phone use) [2]. Smartphone active data can capture high-frequency, in-the-moment subjective assessments of symptom experiences, and the individual-level context of a unique patient’s environment. Smartphone passive information, such as phone calls, text, and app usage, daily activities, and movement data, could reflect proxies of an individual’s daily habits, levels of fatigue, and cognitive disturbance in addition to facets of disease burden and quality of life [3]. Collectively, P-DHTs could detect objective measures of key symptoms that are unique and common across different rare or complex diseases. This rich information can be returned to the user in near real time, and if returned in the correct way, could empower patients to glean new insights into their condition and its modifiers—a “compass” view.

The challenge in the collection of such multimodal objective and subjective information is how to effectively return this back to the user, or draw associations that are insightful [4]. Several consumer brand wearable devices enable users to follow specific measures of health via an associated app [5]. However, there is a lack of user-driven personalization and limited options to combine information from multiple P-DHTs among existing apps, limiting a true multimodal approach to better understanding health. Furthermore, existing apps are largely proprietary-based, come with a paywall, hide algorithms behind provided data and insights, and are not open source. These realities hinder progress to modify and tailor to the individual or population and limit the patient community's ability to benefit from collective insights [2]. An additional challenge is to inspire a user to engage with the app in the long term, so that health-related insights can be made. Most consumer apps target the achievement of fitness goals that are not appropriate for those with chronic illnesses [6-9]. Further, existing health-related apps show strikingly poor engagement [10-13]. Evidence suggests that this low engagement is in part the result of a lack of personalization and, in turn, low perceived value of the tool [1].

Co-design has become a nebulous concept with numerous definitions and applications. While widely used, its applications are underdeveloped, particularly in the P-DHT space [14]. Co-design, sometimes referred to as participatory design, is often described as a method of including end users in a design process to ensure their needs and voices are present to improve the impact of the intended outcomes. However, the level of participation and timing in the design process varies across definitions and applications. Collaborative design considers all participants as experts in their respective domains. User-centered design is an approach that centers on the needs and preferences of the end user through an iterative process in real-world situations during the entire design timeline to better understand when and where a particular health care tool or intervention will work or fail [15]. Patient-centered design aims to empower patients as agents of their own health and emphasizes patient acceptability and usability [16,17], while patient-led design assigns patients the role of partners, as opposed to end users, or collaborators in the design process—another approach aimed at increasing agency in health. The latter two approaches have unique relevance in the context of P-DHTs that could empower patients to take greater control over their health, potentially without the guiding role of the clinician, shifting the power balance between doctor and patients [4]. There are several other approaches used that describe variants of the approaches listed above (eg, human-centered [18,19], collaborative, and person-based [20]).

In the past 5 years, a number of digital health co-design frameworks have surfaced that show promise in the cocreation of P-DHTs for a number of applications, including co-designing a smartphone app to improve access to mental health care [21], interventions for obesity [22], and support for people with dementia [23]. While these modern applications include powerful inclusion of end users from the inception of digital design, challenges remain. The complexity in both the development and use of a P-DHT requires a certain level of digital literacy that can result in roadblocks in empowering end users to co-design [23]. Further, there is still an under-involvement of end users in the final usability stages. The needs and realities of how an end user will want to engage with a personal health app can only be realized through the long-term use of a tool through different health states. Usability testing in current co-design applications is limited, and the definition of what is usable is currently vague.

The objective of this paper is to describe an approach to a user-centered, collaborative co-design process in the development of 5 prototype apps for different end user populations, including individuals with long COVID-19, pancreatitis, sarcoidosis, valosin-containing protein (VCP) disease, and primary ciliary dyskinesia (PCD). These apps were codeveloped as part of the Biohub’s Rare As One Project, which aimed to support co-design prototype symptom tracking and transmitting apps for rare or complex conditions. In this paper, we discuss lessons learned and provide a health app co-design framework for others hoping to develop a personal health app, including app development novices.

## Methods

### App Co-Design Synopsis

**Figure 1:**
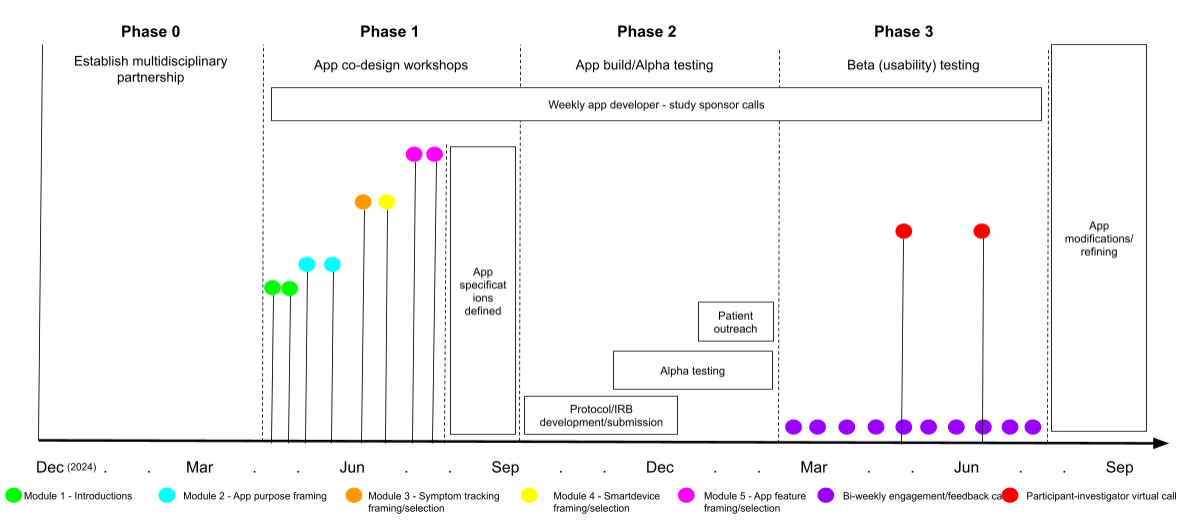
Co-design project methodology timeline.

**Figure 2:**
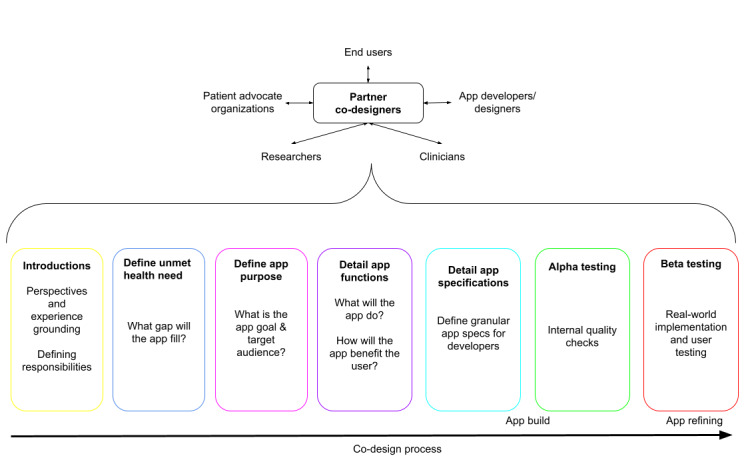
Patient-led, user-centered, and collaborative personal digital health technology co-design.

This project involved a multidisciplinary partnership between the sponsor, 4YouandMe [24] (a nonprofit digital health organization that includes expertise in app design, user experience [UX] and remote digital health app–based research), AgileOps (a software development company), and 5 different disease areas including a patient-driven organization (PDG) lead (patient advocacy groups), an individual with lived experience of the condition (herein referred to as end user), a clinician with condition-specific experience (selected by the PDG), device partners (Empatica, Lumia Health, MIR, and Oura), and Biohub (the study funder). All partners were involved in an extensive 6-month co-design phase (phase 1) that was conducted entirely remotely through frequent virtual meetings and workshops. This was followed by a study preparatory phase to build out the first versions of the prototype apps, prepare institutional review board (IRB) materials, and start patient outreach to identify end user testers (phase 2). Phase 3 involved a 5-month beta testing pilot longitudinal study of approximately 50 participants for each prototype app (Figure 1). The co-design methodology adopted and extended principles from many co-design concepts (user-centered, collaborative, and patient-led), where end users were placed at the center of the design process alongside other equal multidisciplinary partners(Figure 2). We adopted a “by each other for each other” approach where disease experts (end users, clinicians, and PDGs) were not expected to design themselves, but were empowered and provided with the tools to co-design alongside app development experts (app developers, UX experts, and digital health researchers). This approach resulted in significant investment of time from all partners involved. The PDGs received small grants to compensate for their invested time.

### Development of Multidisciplinary Team (Phase 0)

The first project phase involved the curation of the project team. 4YouandMe worked with Biohub to select 5 different PDGs. These groups were selected based on the following criteria: PDGs having motivation and capacity to participate in the patient-led design process, the ability to identify a clinician representative to participate in the co-design, and the ability to recruit 50 participants for the pilot study. The groups were additionally curated to include diverse disease areas, but with some overlap in symptoms, so that insights could be made from each group. The final selected groups included the following: Patient-led Research Collaborative (long COVID-19), Mission Cure (pancreatitis), Foundation for Sarcoidosis Research, Cure VCP Disease, Inc, and the Primary Ciliary Dyskinesia Foundation.

### App Co-Design (Phase 1)

A 6-month app co-design phase was conducted involving the codevelopment of specifications through group workshops and independent exercises developed by the study sponsor that defined the goal, content, features, and UX of each app. This initial phase involved 5 modules (Table 1). The workshops provided an avenue to ground the app development experts in the experience and needs of patients with the disease, and the disease experts in aspects of the app development process. Each workshop included grounding virtual presentations by specific partners depending on the intended outcome, and all included an informal discussion period (Table 1). The co-design process was iterative and bidirectional, where partners were grounded on key concepts in the app development process and were asked to complete independent exercises (Multimedia Appendix 1) that were then reviewed in subsequent workshops to find common ground and build key stepping stones of the app development roadmap. The app development experts synchronously drafted app specification documents in collaboration with the disease experts, which were then used to communicate the final app specifications to the app builders. An example of an app specification document can be found in Multimedia Appendix 2. At each co-design step, PDGs added substantive considerations for end user needs that shaped the app specifications.

This app co-design was guided by core principles, including the following: (1) personal health apps must give back to the end user—what is the app’s gift or benefit to the end user; (2) personal health apps must minimize the risk of instigating negative health outcomes; (3) personal health apps aimed at providing insights on specific diseases must be reflective of the unique needs of the patients in those communities; and (4) a personal health app’s success cannot be fully measured without a significant amount of usability testing.

**Table 1:** App co-design module descriptions.

Module, purpose, and workshops (presenter)				Outcome		
Module 1. Introductions						
	Introduction between partners, description of partner goals, roles, and responsibilities					
		Introduce approach, and app development process (sponsor) What is the current state of the health app market? What are health apps possibilities and limitations? Co-design approach (“by each other for each other”)	Educate disease experts about the app development process, realities			
		Introduce disease (PDGs^a^) What is the disease epidemiology, burden? What are the patient and clinician unmet needs, gaps in care What is the PDGs past work/experience in digital health research app development?	Educate app development experts about each disease, patient, and clinician perspectives. Gauge all partners’ experience in app co-design			
Module 2. App purpose/function framing						
	Co-design primary app goals and key functionalities					
		Defining the app purpose Sponsor synthesis and discussion of PDG’s suggested patient and clinician needs App guiding principles Description of app possible attributes Independent exercise 1-Preference of potential app purpose/attributes	App goal and purpose framed and understood by all partners			
Module 3. Symptom selection						
	Co-design a list of core symptoms to be tracked and transmitted					
		Describing subjective and objective symptom tracking through P-DHT^b^ possibilities and realities (sponsor) What symptoms are apps/wearables able to track well, not well? Challenges in symptom tracking (confounding, uniqueness of individuals, linking objective measures to subjective symptoms) Important of individual context Introducing app-based visual tools for following symptoms, gaining health insights Independent exercise 2-define specific app attributes	Disease experts grounded on app/wearable realities in following specific symptoms and states of health			
		Review and discuss independent exercises 1 and 2 (all partners)	Define app attributes and key symptoms to be tracked and transmitted			
Module 4. Digital tool selection/dashboard						
	Consider which on the market wearable or smart device could enable the objective tracking of the identified key symptoms, and introduce digital dashboards.					
		Introduction to potential digital tool options (sponsor) Grounding on current on the market wearable/smart devices realities and limitations Sponsor digital tool suggestions based on PDGs disease/symptoms Introduction to digital dashboard (ways to follow/share symptoms, experiences) Introduction to triggers/modifiers (context labels) and how they can be captured through dashboards Independent exercise 3-dashboard preferences	Wearable/smart device selected Disease experts grounded on digital dashboard purpose, realities, limits			
Module 5. App feature/UX^c^ introduction/app attribute refining						
	Determine specific surveys/active tasks, or develop new tasks to enable the subjective tracking of the identified key symptoms, and introduce and refine app features and functionality, and UX.					
		Introduction to potential app features and UX/app specification review (sponsor) Introduce possible app features (beyond surveys/tasks) to capture symptoms and context Brief introduction to app UX Provide and review app specification documentation Independent exercise 4-full app specification review	App content (surveys/tasks) fully defined in the form of an app specification document			
	1 on 1 UX session to finalize app feel, and feature function					
		1 on 1 with UX expert (UX expert) Introduction to app UX Sponsor synthesis and discussion of PDG’s suggested features Independent exercise 5-UX preferences	App UX defined			

^a^PDG: patient-driven organization.

^b^P-DHT: personal digital health technology.

^c^UX: user experience.

### Study Preparatory Phase (Phase 2)

A 5-month study preparatory phase was conducted to prepare for phase 3 of the co-design (a pilot beta testing study with up to 50 end user participants for each prototype app). During this phase, the technology partner built the first version of each app, while in parallel, IRB materials (protocols, consent forms, and participant-facing materials) were codeveloped with all partners and submitted for approval, wearable/smart device companies were engaged, and agreements were completed. During the second half of this preparatory phase, internal alpha testing was conducted by the study partners, and patient outreach began to identify end user participants for each prototype app. Monthly one-on-one virtual meetings were conducted to give disease experts updates on app development and to co-design IRB documents. This period also included an alpha testing phase, where all partners performed an initial usability testing of the app to identify initial bugs before opening up the app to participants.

### App Optimization—Beta Testing Pilot Study (Phase 3)

#### Overview

A 5-month pilot study was conducted with US-based end user participants to perform initial usability testing of the app. Study advertisements were co-designed with each PDG. Participants were recruited through each PDG organization using social media and newsletter advertisements (see Multimedia Appendix 3 for an example) with a cover letter from each PDG introducing the pilot study. Participants were instructed to contact a 4YouandMe study representative and complete an initial screening phone call where they were explained the study, their eligibility was confirmed, and they were given instructions on how to download the app. Briefly, participants were eligible if they were 18 years or older (except for the PCD beta testers that included a minor version of the app, inviting 14-17 year olds), had a suspected or clinician-confirmed diagnosis of each disease, and owned a personal iPhone/Android smartphone (see Multimedia Appendix 4 for full inclusion criteria). The app included an e-consent where participants officially agreed to participate, including a parental authorization mechanism for participating minors (PCD app). Post enrollment, participants were mailed their cohort-specific wearable/smart devices. Participants completed participation in an entirely remote setting.

#### Beta Pilot Study Procedures

Enrolled participants were instructed to download the prototype app, provisioned with specific wearable and/or smart devices (Multimedia Appendix 5), and instructed to use these devices continuously during their participation. Participants were asked to complete biweekly phone check-in calls with a dedicated engagement specialist. These calls aimed to support participants, work through technological issues, and collect their feedback as beta testers. Engagement specialists entered participant feedback into structured and unstructured fields in Research Electronic Data Capture—a secure data management database. Participant feedback was documented continuously and systematically by engagement specialists, centering on the following themes: functionality, usability, harms, benefits, self-explorations, and others (Textbox 1). Participants were also invited to participate in 2 optional virtual investigator-participant meetings where they had a chance to meet the study team, get study updates, and ask the study team questions in real time, anonymously.

**Beta testing app themes collated from participants.**

**Functionality**

- Are the study tools (study app, wearables, and smart devices) functioning the way they should based on their intended use—to enable the user to track diverse symptoms and modifiers of their condition and potentially provide insight into factors that impact symptoms and outcomes, or general health insight relating to their condition. Are there factors about the participants’ conditions that are not being tracked that should be? Are there missing functionalities that could help achieve the study tools goals?

**Usability**

- Are the study tools usable? Are there internal or external factors that prevent the participant from using the tools for their intended use (eg, physical barriers such as mobility challenges, mental barriers such as fatigue or cognitive disturbances, and technological barriers such as WiFi connectivity)?

**Benefits**

- Are there reported benefits from using the study tools? Do participants perceive value in using the study tools? Benefits might include factors such as improved health insight and awareness, or positive behavior modification.

**Harms**

- Are there reported harms from using the study tools? Harms might include factors such as increased anxiety or other mental health concerns from tracking certain symptoms or being asked certain questions at high frequencies, irritability from task burden, or injuries from using the wearable/smart devices.

**Self-explorations**

- How are participants using the app to learn about themselves and conduct self-explorations? How specifically are they using the compass feature? For example, are they using this feature to track specific symptoms/features, to track specific modifiers of symptoms (eg, diet), or to try to learn associations between factors that impact symptoms?

### Ethical Considerations

This study was approved by Advarra IRB (Pr00082545) and all participants digitally provided informed consent and parental authorization (where appropriate). All personal data were kept confidential by the internal research team. The study data collected were stored in deidentified form (stripped of personal identifiers) and attached to a unique identifier. Participants received US $50 for every month of participation in the form of a gift card and were also offered to keep the Oura ring at the end of the study.

### Prioritizing Participant Feedback

Participants communicated their feedback to their engagement specialists through text, email, video calls, and, where appropriate, sent screenshots. Participant feedback was documented systematically by engagement specialists, which was then collated by the app development team and reviewed with PDGs for prioritization. This project had time and funding constraints, meaning that not all participant suggestions could be implemented. Suggestions were condensed from duplicates across participants, and by setting aside those that were out of scope (ie, could not be achieved within the current time frame, or within the current budget). PDGs were asked to rate each feedback from least desired, desired, to most desired. The app development team worked to determine which of the desired and most desirable suggestions could feasibly be implemented within the allotted time frame and budget. This synthesis was conducted in 2 waves during the beta study, with the goal of having time to implement most suggestions so that the participants could view and test themselves. Suggestions that were not possible to implement were documented and are made available to others wishing to view (see Data Availability section).

### App Development Process

App development was undertaken by the technology partner (AgileOps) using an iterative, Agile workflow aligned to the multiphase co-design program. Requirements were derived from phase 1 specification documents and UX exercises, translated into shared low- and high-fidelity Figma prototypes for rapid validation with the project team and ultimately all the stakeholders involved in the co-design exercise, and then implemented in short, time-boxed iterative development sprints (recurring unit of time by which the development process is organized) of an approximate length of 2 weeks each with traceability to the originating workshop artifacts. Every app change was mapped to a requirement or user story traceable to co-design artifacts (workshop outcomes, spec docs, and Figma frames). Weekly product reviews with PDGs and the sponsor confirmed scope, prioritized participant-reported issues, and aligned releases with IRB amendment needs. All 5 study apps were developed natively for iOS and Android and distributed via the Apple App Store and Google Play Store. The supporting backend services were deployed on cloud infrastructure to enable secure survey delivery and wearable data acquisition. Continuous integration and delivery followed trunk-based development with mandatory code review, automated linting and tests, and staged promotion (development → staging → production). Mobile releases used internal/beta channels for rapid usability checks prior to public availability during phase 3. A server-driven “study engine” delivered survey schemas, schedules, notifications, and visualization configurations by remote configuration, allowing same-day adjustments while preserving data integrity through schema versioning. This engine enabled real-time incorporation of participant feedback during active use.

In terms of accessibility and performance, frontends follow web content accessibility guidelines–informed patterns (contrast, type scaling, and motion reduction). Mobile apps are optimized for low-bandwidth scenarios with appropriate error and conflict handling. The Compass Chart experience specifically used progressive data loading and elastic queries to keep visualizations responsive even as longitudinal data grows.

Quality and safety practices included a testing pyramid (unit, integration, and end-to-end), synthetic monitors of critical user journeys (enrollment, consent, survey completion, and device synchronization), centralized observability (logs/metrics/traces), and auditing of administrative and export actions. All network communication used transport layer security; storage and secrets followed cloud key-management and least-privilege access controls. These measures supported the usability study while meeting governance needs associated with IRB-approved procedures.

### Architectural Stack and Methodology

A key element that facilitated the continuous development process was the methodological and technological stack adopted for the project, which was intentionally fine-tuned for quick prototyping, iterative development, and continuous release without compromising the quality and security of the output. This was achieved by combining modern development frameworks (specifically Ruby on Rails application layer exposing versioned application programming interfaces), web consoles for research/clinical use, and native iOS/Android apps for participants to support robust device integrations and accessibility features, all backed by a cloud-first infrastructure containerized and orchestrated on Amazon Elastic Kubernetes Service (Kubernetes) to support isolated workloads, horizontal scaling, and controlled rollouts. Data from surveys, active tasks, and connected devices were normalized through event-driven extract, transform, load pipelines and indexed in Elasticsearch to enable longitudinal visualization and query of symptoms and objective signals.

### Analysis

Phase 1 of this co-design involved building out app specification documents. All of the resulting app prototype themes/attributes and outcomes are described in tabular format. Participant insight and feedback from phase 3 were collected through regular phone discussions every 2 weeks with study engagement specialists, in addition to an exit interview after completing the study. A thematic analysis [25,26] of qualitative data captured during these interactions was conducted to identify themes for app improvement. Participant engagement was measured through calculating retention rates (number of dropouts/total enrolled) and median adherence to study activities over study follow-up. Adherence to app-related tasks was defined as the proportion of tasks completed divided by the total number of tasks that could have been completed over each participant’s unique study follow-up. For example, all apps included a daily quick activity. For a participant with a follow-up of 4 months, the total expected number of daily quick activities is 120. If a participant completed 95 daily quick activities, their adherence to this specific task would be 79%. Adherence to the check-in calls every 2 weeks was defined as the number of calls completed divided by the total number of expected calls over a unique participant’s follow-up period. Medians and ranges were used as adherence distributions were nonnormally distributed. Adherence to the wearable/smart devices was estimated using different daily wear criteria. For Oura ring data, daily wear was defined as the presence of an “Oura Sleep Score.” For the Lumia device, daily wear was defined as the presence of at least one “heart rate” value. For the Empatica device, daily wear was defined as the presence of at least one “electrodermal activity (EDA)” value. For the MIR Spirometer, daily wear was defined as the recording of any spirometry data.

## Results

### Co-Design Outcomes

Multimedia Appendix 6 outlines the codeveloped app goals and specifications for each prototype app. While there was overlap in unique goals, themes, and key symptoms to track across the prototype apps, each app included unique content. Each group had an interest in building longitudinal symptom maps and having the ability to share symptoms with clinicians. Further, all groups had an interest in enabling the user to follow aspects of burden of disease, routines, and potential triggers that modify symptoms or disease outcomes. This stage in co-design was critical for deciding on the prototype app's key functionalities and features to ensure that the tool could achieve the common and specific objectives. While most groups included a target age range of >18 years, given the mostly adult onset of symptoms, the PCD app included a minor app version (aged 14-17 years) in light of the presence of symptoms earlier in life.

Select wearable/smart devices were selected for each group to capture objective measures of key symptoms (section 4B in Multimedia Appendix 6). All groups used the Oura ring as a measure of daytime activity and sleep quality. Groups that had a specific interest in following the symptom of pain additionally included the Empatica EmbracePlus smart wristband device, owing to its ability to track EDA (skin conductance), which could reflect an objective proxy of pain. Additionally, the long COVID-19 group had an interest in following dysautonomia (dysregulation in the autonomic nervous system), for which EDA could be a useful measure. The Lumia ear device was additionally used in the long COVID-19 group owing to its unique ability to capture blood flow to the head as another useful potential measure of dysautonomia. Groups that had an interest in following symptoms relating to breathing, or respiratory function, additionally included a spirometer (MIR Spirobank Spirometer). Please see Multimedia Appendix 5 for a detailed description of selected wearables and smart devices. Taken together, other than design and targeted symptoms (which defined the app survey/task content and wearable/smart device selection), the apps were similar in design with the same core features.

### Prototype Apps and Their Features

**Figure 3:**
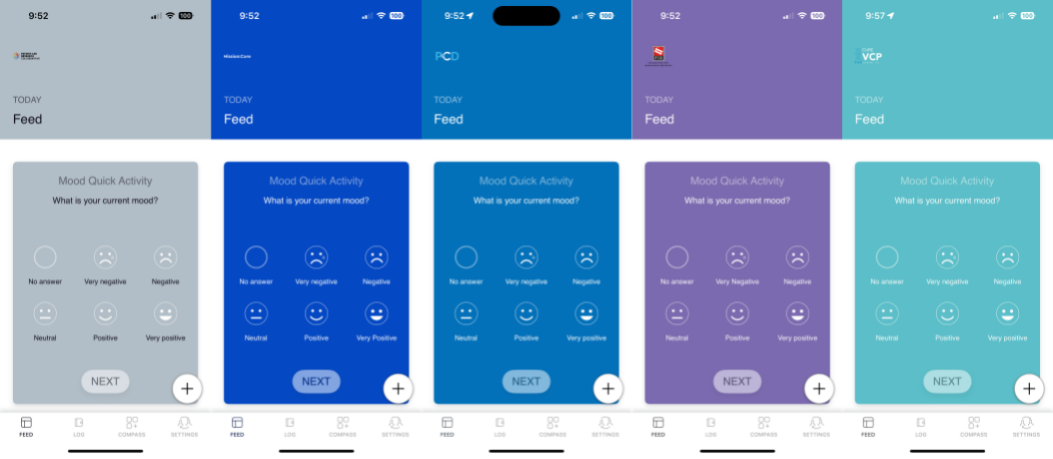
The prototype apps.

Specific app features were developed to accommodate the intended app themes and goals. All prototype apps included daily and intermittent prompted surveys and active tasks to collect subjective self-reported information from participants that were intended to capture each symptom in order to build longitudinal symptom maps across conditions (Figure 3).

In keeping with many group themes of enabling better abilities to track and understand certain symptoms and have the ability to better understand triggers and modifiers of symptoms and disease, additional optional app features were co-designed. These included the “I’ve Noticed...” feature, the “Reflections” feature, the Compass Chart, and Compass Log.

The “I’ve Noticed...” feature (section 5A in Multimedia Appendix 7) enables the end user to tag momentary experiences at any time in the app in either a written text format, or audio or video recording. The logged experiences from the I’ve Noticed feature are then time-stamped and logged in an additional diary format feature called “Compass Log” (section 5B in Multimedia Appendix 7), where the end user can return to these logged experiences and even edit them. Participants were instructed to try to use this feature to log in-the-moment potential triggers/modifiers that could help explain fluctuations in their symptoms, or for logging dynamic changes in their symptoms. For example, for the VCP group, this feature was useful for logging falls and near falls when they occurred. This feature was developed to enable a user to tag momentary context in their day or night that could lend insight into potential triggers or modifiers of their condition.

The “Reflections” feature (section 5C in Multimedia Appendix 7), while similar in format to the “I’ve Noticed...” feature, served a different purpose. The “Reflections” feature was designed to empower the user to have a space within the app to take the time to consider certain aspects of their condition and reflect on certain aspects that improve or deteriorate their condition. This feature prompted participants daily to complete a reflection where they could write a written note, or complete an audio or video recording. Prior to completing a reflection, this feature instructed participants to complete a grounding exercise, such as meditation, going for a walk, etc, to help prepare themselves to make a reflection about their day or their week in relation to their condition. The logged reflections were made available in the end users’ Compass Log, where they could revisit their logged entries. This feature was developed to empower the end user to reflect on a deeper level of possible triggers and modifiers of their condition.

**Figure 4:**
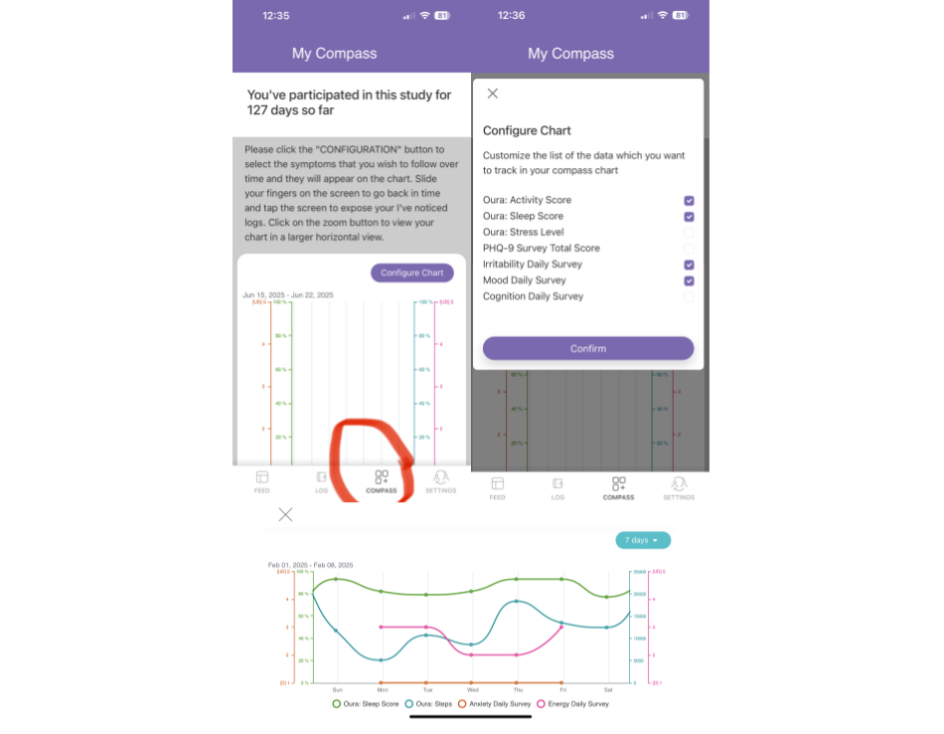
My Compass Chart feature.

The Compass Chart (Figure 4) feature enabled participants to view select symptoms or wearable/smart data streams retrospectively over time. Participants could select from a dropdown menu of up to 10 items to track and were able to view 4 of these items in their chart at a time. This feature was designed to provide the user with a tool to follow, and potentially learn new insights about their condition in terms of symptom patterns and factors that improved or worsened their condition.

### Beta Testing Pilot Study Results

#### Overview

A total of 239 end user participants participated in the beta testing pilot study that was conducted between February 2025 and June 2025. See Multimedia Appendix 8 for specific enrollment flow details of each group. Enrollment and retention rates were high, ranging from 94% to 100% and 92.2% to 100%, respectively (Table 2). In most disease groups, exit interview completion was high (long COVID-19: 47/50; pancreatitis: 35/44, 80%; sarcoidosis: 44/50, 80%; VCP: 42/44, 95.4%) except for the PCD group (23/51, 45.1%), who were mostly minors. Demographic characteristics can be found in Multimedia Appendix 9. In all but the sarcoidosis cohort, beta testing participants were predominantly White and female. Mean age was in the middle age category (40-55 years) in all groups except for the PCD cohort, which included minors, with a mean age of 31 (SD 15) years.

**Table 2:** Median adherence rates for study activities across disease groups.

	Long COVID-19 (n=49)	Pancreatitis (n=44)	PCD^a^ (n=51)	Sarcoidosis (n=50)	VCP^b^ disease (n=44)
Study weeks, mean (SD)	14.2 (3.6)	14.5 (3.2)	11.9 (3.5)	18.0 (0.6)	13.7 (3.1)
Retention, n (%)	49 (100)	42 (95.5)	47 (92.2)	47 (94.0)	44 (100)
Biweekly check-in calls (%), median (IQR)	100 (100-100)	85.7 (61.7-100)	71.4 (46.4-92.9)	85.7 (71.4-100)	100 (100-100)
Daily quick activities (%), median (IQR)	36.8 (22.1-60.3)	39.9 (23.5-68.2)	39.8 (24.2-55.4)	51.2 (25.1-67.3)	43.1 (29.7-64.2)
Weekly in-app survey (%), median (IQR)	90.9^c^ (73.3-100)	97.2^c^ (89.2-100)	100^c^ (80.2-100)	—^d^	72.4^e^ (54.2-80.0)
Biweekly in-app survey (%), median (IQR)	100^f^ (75-100)	100^f^ (88.9-100)	100^f^ (79.2-100)	100^f^ (89.5-100)	100^f^ (100-100)
Oura ring use (%), median (IQR)	89.3 (71.4-95.0)	67.1 (46.2-92.5)	26.3 (15.5-50.4)	66.4 (52.6-82.1)	83.9 (65.1-94.1)
Empatica Embrace Plus use (%), median (IQR)	69.9 (39.4-98.2)	69.7 (29.9-93.1)	—	—	—
MIR Spirometer use (%), median (IQR)	—	—	50.0 (33.3-62.5)	—	62.5 (47.2-81.7)
Lumia use (%), median (IQR)	62.5 (33.7-80.0)	—	—	—	—

^a^PCD: primary ciliary dyskinesia.

^b^VCP: valosin-containing protein.

^c^Weekly Fatigue Assessment Scale.

^d^Not available.

^e^Weekly Pain Severity Scale.

^f^9-item Patient Health Questionnaire.

#### Participant Adherence

Table 2 includes median adherence to study activities by disease group. With the exception of the PCD group, all groups demonstrated excellent adherence to participating in biweekly check-in calls with research staff and to weekly and biweekly in-app surveys. Adherence to using the study smart devices demonstrated acceptability, with participants using the Lumia ear device >60% of the time, Oura ring use >65% of the time (except for the PCD group), Empatica use >55% of the time, and MIR Spirometer use >50% of the time. All devices except the Oura ring had a delayed integration into the study, meaning that the time that participants had to interact with these devices was shorter than the entire study duration. The PCD group was the only group that included minor participants (aged 14-17 years). This disease group demonstrated consistently lower adherence to most study activities.

As this was a pilot beta testing study, the app was undergoing modifications, and a number of active tasks and features had periods of malfunction. Accordingly, the adherence estimates relating to the app tasks should be interpreted with caution, as some of the low adherence may be attributable to app error as opposed to noncompliance.

#### Participant Beta Tester Feedback

During the participant beta testing phase, 215 unique participant reports of prototype app issues were documented, while 112 unique participant reports of wearable/smart device issues were reported. There were 257 unique participant suggestions of how to modify or improve the study app experience. User feedback was coded into the following core themes: 81 functionality, 51 usability, 28 benefits, 20 harms, 71 self-explorations, and 6 others. Several features were implemented during the beta testing period, and some shortly after the study closed, based on participant feedback. For example, new surveys or new items to existing surveys were included, screening questions, and features to instruct participants not to complete tasks when they cannot safely be completed (eg, walk tests) to ease user burden, color contrast improvements, including a dark mode option for users with visual impairments, labeling features with emojis to improve ease and personalized “I’ve noticed”/”reflection” entries. See Multimedia Appendix 10 for all features implemented.

Over 90% (175/191) of participants across all disease groups (who completed an exit interview) reported that they received some benefit from using the prototype apps and associated wearable/smart devices (long COVID-19: 42/47, 89.4%; pancreatitis: 34/35, 97.1%; sarcoidosis: 41/44, 83.2%; VCP: 38/42, 90.5%; PCD: 20/23, 87.0%). Overall, 71.2% (136/191) of all participants rated their likelihood to use their respective app in the future between 7-10, on a 1-10 rating scale, with 10 being the most likely to use the app (Multimedia Appendix 11). Participants reported benefits of the prototype apps and associated wearable/smart devices, including increased self-awareness of symptom modifiers (eg, sleep and stress patterns that trigger exacerbations) and behavior modification through the encouragement of health-promoting behaviors. Participants indicated an appreciation for the comprehensive symptom tracking that empowered them to feel accountable for making healthier choices and the desire to share their health data with others. Participants also reported that they felt supported in participating in the beta testing study. Frequent check-in calls with engagement specialists and intermittent virtual progress meetings with the investigator team were viewed positively by participants.

While benefit was reported by the majority of participants, a number of subthemes emerged during the beta testing usability period (Multimedia Appendix 12). There was a desire to improve the functionality of the apps through better personalized notifications when tasks are due, better task instructions, and better app navigation through the app’s core features. There was some overlap in feedback between the core themes of functionality and usability, where, for example, more personal customization was a desire, which alters initial functionality, but would likely lead to better usability. Numerous participants had different opinions on how frequently they wanted to complete certain surveys. For example, all prototype apps included a daily quick activity survey that prompted the user to report on their mood, energy, cognition, pain, and other symptoms. A number of participants suggested they track these symptoms multiple times a day to capture daily variations in fluctuating symptoms, while others preferred a longer cadence (eg, weekly), and some participants suggested tracking additional symptoms, not present in the quick activity. A solution would be to enable participants to set their own cadence and even the content of some surveys. However, in making these decisions, one must weigh the consequences of enabling too much customization where data cannot be harmonized across users for health research, or returned data are no longer useful for the individual. There were unique usability preferences from the long COVID-19 group, many of whom reported vision problems. These reports included enabling a dark mode option or better color contrast.

There were 20/239 (8.4%) unique participant reports of nonserious harms associated with using the prototype apps and associated wearable/smart devices (10 long COVID-19, 5 pancreatitis, and 5 VCP). Most of these were related to frustration relating to unclear instructions on certain tasks and having to complete tasks that did not feel relevant to the user. Many of the prototype apps used select ResearchKit’s active tasks (eg, 2-minute walk task). Many of these “plug-in” tasks are not customizable, which introduces challenges to include adequate instructions to tailor these tasks for each end user population. There were also some reports of burden and fatigue from too many tasks, particularly when symptoms were static. A small number of participants reported a negative emotional impact from using the prototype apps and associated wearable/devices. For example, there was a report that the use of the Oura ring and returned summary scores made a participant feel worse about their health (VCP participant). For some, persistent, daily tasks felt like reminders of disease progression and were emotionally draining.

In addition to these reported harms, there were several reports of skin irritation issues associated with wearing the Empatica EmbracePlus wristband at the site of wear that appeared to be mostly the result of contact dermatitis. This was investigated with the device company, and Empatica has reported 0.1% skin irritation rate for EmbracePlus, according to tests performed following the ISO 10993 standard series. Both of the populations using this device may experience heightened autonomic activity from pain (pancreatitis) and from other unknown causes (long COVID-19) that could have contributed to a higher number of reported irritations than typical for this device. Participants were instructed to stop wearing the device if skin irritation occurred.

Participants were encouraged to use the prototype app Compass feature to log experiences and symptom modifiers, and use the Compass Chart to track certain symptoms over time. This feature also overlaid select feature streams from some of the wearable/smart devices. The goal of this feature was to enable the end user to start to self-explore their condition, and potentially start to make some insights into what makes them better or worse. There was again overlap in feedback themes, with this core theme relating to the desire for customization in what participants were enabled to track via the app, and how this was transmitted back to them via the app. Participants’ desires for what and how to transmit via the app were heterogeneous. However, there was a general consensus that personalized labeling (eg, using icons and color coding) within the Compass Chart would be useful, and ways to export and share data with others for a deeper review of personal health data.

#### PDG Co-Designer Experience

The participating PDGs reported positive experiences relating to being able to shape an app, learning from other PDGs and from an app development team, exploring the use of P-DHT data that is important to patients, and providing patient communities with the opportunity to pilot test an app for them.

Negative experiences were reported relating to mismatches in project objectives. Some of the PDGs anticipated that the data could be used to inform insights about each condition or the use of the tools for following certain symptoms—there was an expectation that direct benefit would be translated to the participating communities. This highlights the critical importance of repeating and discussing project objectives throughout the entire co-design process. To effectively co-design and beta test a prototype app, end users must use and interact with the app, which means data is produced. However, with dynamic changes occurring within the app, and the resolving of early app bugs that can result in significant gaps in data, the data produced from such early prototype stages is not advisable to be used for making health-related insights that one would make from an observational study.

PDG groups were heavily involved in the first 2 project phases, but were less involved in phase 3—the beta testing study, which was more focused on end user involvement. There was a general desire from PDGs to be more involved in the beta testing study to relay updates back to their community, and to better understand insights from the data in near real time.

Some PDGs reported wanting more one-on-one time with the development team. A number of group workshops were held, but all co-designers felt that more time was needed to work through questions and complex ideas.

#### App Development Team Co-Designer Experience

The app development team found benefit in the co-design approach in producing a prototype app that could have taken multiple usability studies to achieve. The initial PDG input on app goal and specifications was instrumental in yielding an early prototype that already had elements of disease-specific personalization. This allowed the beta testing period to focus on more nuanced features of the app as opposed to common user feedback on relevance. In the engagement of ~50 end user beta testers for months, the sheer volume of detailed app improvement input was overwhelming, much more than the app development team has experienced from past P-DHT studies. Further, while the app development team has reported high retention and adherence rates from past P-DHT studies [1], the observed enrollment and retention rates of end users in this pilot study were exceptionally high.

The app development team underestimated the time and resource burden of the co-design approach, especially in the development of 5 different apps. There was an expectation that more overlap in needs across the disease groups would be present; however, the initial co-design phase highlighted each group’s unique needs, which surpassed the app development team’s initial expectations.

## Discussion

### Overview

In this paper, we describe a patient-led, user-centered, and collaborative personal health app plus wearable plug-in co-design methodology. Five prototype apps were developed for end users with long COVID-19, pancreatitis, sarcoidosis, PCD, and VCP disease. End users were placed at the center of the design process alongside other multidisciplinary partners driven by nonprofits and community-based organizations, including app design and development experts, UX experts, clinicians, and PDGs. Using a “by each other for each other” approach, this co-design process included facilitators to guide the co-design process for disease experts; however, all partners were considered experts in the process in their own respective roles. After the development of the prototype apps, this approach included a 3- to 5-month usability testing period with 239 end users across different rare or complex conditions. A flexible study engine was built that facilitated real-time and frequent app modifications while the app was in active use. This flexible study engine accelerated and empowered the co-design process.

While this co-design methodology required significant effort and time from all partners involved, it yielded deep insights into end user driven prototypes. Beta tester participants demonstrated exceptionally high enrollment rates, retention, and acceptable adherence to using the apps and devices for several months during the usability testing period. A total of 16 of 191 participants actively engaged in providing feedback on biweekly check-in calls, exit interviews, and virtual calls, while 327 unique P-DHT bugs and 257 unique suggestions to improve the app were reported. Collectively, participant suggestions resulted in numerous app fixes and improvements, many of which were implemented while participants were actively using the app, enabling them to see the result of their feedback. Further, pairing participants with an engagement specialist offered a mechanism to communicate complicated app/wearable-related concepts, which would not be possible without these frequent touch points.

While the impact of the co-design approach cannot be formally evaluated from this study, as there was no comparison, the app development team reported a more streamlined process in producing an early personal health app prototype that already included disease-specific personalization. This enables a deeper beta testing focus on more nuanced features of the app. Through both the engagement of the end user participants from the PDGs and the results of the initial co-design stage, the volume of app improvement suggestions was much more than the team had experienced from past P-DHTs studies.

A common participant feedback theme centered around customization of what the app tracked, how often it tracked, and how it transmitted this information back. Further, participants expressed a desire for more instructions and tailoring of certain tasks so that they were relevant to them and their situation. For example, in the VCP and long COVID-19 end user groups, some users experienced phases where they could not walk safely and expressed a desire to temporarily turn off certain app activities (eg, the walk test) so that they were not repeatedly prompted to complete them. A challenge for the co-design team in implementing participant feedback is balancing this feedback with the personal health apps’ intended goals. If the app is purely for the participant, then the co-designer may increase the level of customization. However, if information from the app is intended to be shared with others (eg, clinicians or researchers for analysis), the co-designer has to balance the level of customization with the level of data harmonization that is required to meet the app’s goals. This highlights the continued importance of repetitive grounding of the P-DHT intended goal and use for all co-designers involved.

The information collected from P-DHTs holds promise in providing clinicians with richer, higher-resolution data of their patients’ health in between health care visits and new knowledge for PDG research. However, a key promise is in empowering patients with the tools to follow their health, and gain insight about what makes them better or worse, in their own unique context, without the sole reliance on health care visits [2,4]. This could be enabled by augmenting P-DHTs with artificial intelligence (AI) tools that give n-of-1 suggestions for improved health [27-29]. AI-driven insights from P-DHT n-of-1 data could yield new insights into rare or complex disease symptom trajectories, and modifiers of disease exacerbations that account for individual-level variability. However, challenges exist in enabling AI suggestion engines to work effectively. This suggestion engine requires adequate “label” context data from the user, which is notoriously difficult to capture, as it often requires a high burden from the user, and much user validation work is needed to determine the accuracy of these tools’ suggestions.

There are additional challenges in the general use of P-DHTs. P-DHTs involve a certain level of digital literacy and usability to be feasible for use in the long term and integrated into patients’ daily lives. Further, there are disconnects in values and approaches to bring a P-DHT from design to implementation, resulting from multiple stakeholders between health care and industry that often leave the end user at the outskirts of the process [14]. Finally, the existing health app market is saturated by companies with pay walls, and that hold data in silos that can result in a health data market that takes advantage of patients and their sensitive data. Taken together, P-DHTs hold promise to increase patient agency, putting them in the driver’s seat of their health. However, for this promise to become a reality, patients and community organizations must first be empowered and given the time to cocreate these tools for themselves, backed by the appropriate companies.

A number of robust P-DHT patient-centered and patient-led co-design approaches have emerged in the past 5-10 years [21-23,30,31]. A common challenge across these instances is how to adequately ground an end user (especially in a remote co-design environment) so that they are empowered to co-design along with other partners with expertise in app design/development, and digital health research [23]. Further, most existing frameworks do not extend the co-design period into the usability testing period and tend to use small user testing groups for short periods of time [12]. The value and shortcomings of a personal health app cannot be realized until significant usage, which is a key shortcoming of existing P-DHTs on the market and is likely in part the result of poor engagement after the first few weeks of use [11,13]. Accordingly, as opposed to using a mobile app rating scale as a fixed metric of user acceptability and usability, which is commonly applied in other app co-design approaches, we implemented a dynamic feedback system that incorporated user feedback over several months.

### Limitations of Approach

The personal health app co-design approach implemented here yielded several lessons learned. While 6 months was allotted for the initial co-design to build out the first app prototypes that included 8 workshops and several break-out meetings, more time was needed to ground and work through complex processes. Some workshops could have been split into 2 or 3 additional workshops. Further, there were occasional mismatches in co-design objectives throughout the project period. For example, the objective of this co-design was to produce prototype apps and conduct usability testing to determine their initial feasibility, and while that objective was clearly communicated at the start of the process, there was disappointment from some PDGs that the pilot data could not be used to yield new information about the disease or to inform clinical decision-making. Revisiting co-design objectives more frequently and understanding stakeholder goals (and limitations) would be useful for grounding all partners back to the same objective.

This co-design process took a significant amount of time and effort from all partners (>1 year with considerable full-time equivalents from some partners). While this can be seen as a limitation, it may be the reality of co-designing a meaningful personal health app. This needed investment should be clearly defined at the outset to manage expectations appropriately. However, this up-front investment could yield a shorter time to implementation. Further, there was a benefit in the ability to have conversations across five different PDGs, where different disease areas were able to learn from one another.

Each app was iteratively modified, even during the beta testing usability period. This approach is uncommon in app development but has value by enabling the end user to see the result of their feedback and further usability testing of a new fix or improvement in real time. A consequence of this approach is that the release of a new or modified feature by a change in code can inadvertently impact functionality in another app feature. While this scenario occurred on a few occasions, the app development team anticipated this reality and had the appropriate resources ready to troubleshoot these inadvertent app bugs when they arose. In the end, this did not significantly impact the co-design timeline and demonstrates that an app does not necessarily have to be locked for modifications during active use. A further challenge with the aforementioned real-time modification approach is the need for frequent research ethics board modifications to amend study protocols, which, depending on the review board, can be associated with time delays and additional costs.

While the initial app prototypes offered benefit to many of the end user testers, there was a proportion of end users (8%) who did not report any benefit. There will likely always be a proportion of individuals who will not receive benefit from a P-DHT–delivered tool for health. A challenge for app co-designers is in defining the success of the personal health app and understanding which patients are most likely to engage and find benefit. In the context of the P-DHT space, broad benefit across a user population may not be a reality, and success may be defined under a different lens relating to narrower n-of-1 outcome definitions.

As evidenced by the low adherence to most tasks in the PCD group, there were challenges in engaging a minor population compared to an adult population. This cohort had a significantly lower completion rate of biweekly check-in calls (<40% of calls), compared to up to 100% for most other groups. This check-in call has been found to be essential in maintaining engagement in the participation in digital health research activities [1]. Minors may require alternate methods of checking in beyond a phone call. Further work is needed to better understand how to engage a youth population in the use of P-DHTs for health. As the sample size in each group was ~50 participants, the numbers were not high enough to examine differences in usability, adherence, or other feedback by factors such as sociodemographic characteristics. However, with the exception of the Sarcoidosis cohort, all beta tester participants were predominantly White. Therefore, these co-design outcomes cannot generalize to diverse populations.

Importantly, the initial purpose of the Rare As One project was to develop at a high level symptom tracking and transmitting apps. In light of this, the broad goal of each app was loosely already defined, and the grounding exercises were designed to aid in tailoring the goals for each unique disease group. For others wishing to use a similar framework, the goal framing workshops may include different grounding exercises (eg, tools that provide access to care or health knowledge) than those configured here.

### Future Directions and Conclusions

This user-centered, collaborative, co-design work was intended to yield open-source code and usability data for 5 prototype apps for long COVID-19, pancreatitis, sarcoidosis, VCP disease, and PCD. Our aim is to enable others to leverage this open-source work (all available via GitHub) to use the built study engine, prototype apps, extract specific features, or modify it for the same or different clinical populations. It is our hope that other PDGs, funders, or commercial entities might leverage these open-access tools, and if not, that this framework might be used and modified for additional use cases. More beta testing and co-design will likely be needed to further evaluate the potential benefit these apps could yield for end users.

Personal health app co-design is rooted in a complex digital landscape that requires a significant amount of up-front effort and time. Despite the up-front investment in time, taking this co-design approach may yield shortcuts in the longer-term implementation of a personal health app. For groups willing to provide this up-front investment, here we provide a co-design framework and the building blocks of five prototype personal health apps that could be leveraged for improving understanding of, and communicating symptoms of, rare or complex diseases. This reflects a potentially novel resource for PDGs and communities actively engaged in research. The ultimate goal of this personal health app co-design is to develop n-of-1 suggestion tools that could empower end users to glean insight into what day-to-day factors improve or exacerbate their condition, which could be transformative for personalized health care, particularly in the rare or complex disease landscape.

## Appendix

Multimedia Appendix 1 Phase 1 co-design activities.

Multimedia Appendix 2 App specification document example (pancreatitis app).

Multimedia Appendix 3 Sample beta testing study advertisement.

Multimedia Appendix 4 Eligibility criteria of beta testing participants.

Multimedia Appendix 5 Wearable/smart device descriptions.

Multimedia Appendix 6 App goals and specifications.

Multimedia Appendix 7 Prototype app features.

Multimedia Appendix 8 Enrollment flows.

Multimedia Appendix 9 Demographic characteristics of beta testing participants across cohorts.

Multimedia Appendix 10 App features implementd from direct participant feedback.

Multimedia Appendix 11 Proportion of participants rating their likelihood to use the app in the future on a 1 (least likely) to 10 (most likely) rating scale.

Multimedia Appendix 12 Participant beta testing feedback themes—thematic analysis.

## Abbreviations

AI: artificial intelligence
EDA: electrodermal activity
IRB: institutional review board
PCD: primary ciliary dyskinesia
PDG: patient-driven organization
P-DHT: personal digital health technology
UX: user experience
VCP: valosin-containing protein

## Data Availability

Among participants who opted in, coded study data (deidentified) are made available on the Synapse platform [32] at Sage Bionetworks [33] and other platforms to be announced with 4YouandMe’s website [24] and can be freely accessed by any researcher who becomes “qualified” by becoming a registered and certified Synapse user [34], and by meeting the specific conditions of use that require submitting an intended data use statement alongside an institutional review board–approved protocol. The specific Synapse Project page can be found on [35] among registered Synapse users.

Source code for the prototype apps is available at the following reproducibility Github link and website page: iOS shared codebase [36], Android shared codebase [37], and web codebase [38].

The investigators encourage others to access, modify, and build upon these open-source prototype apps.
