# Supplementary material for: A Personal Health App and Wearable Co-Design Framework for Rare and Complex Diseases: User-Centered, Collaborative Co-Design Study
Source: JMIR Mhealth Uhealth. 2026 Jul 20;14:e85425. doi: 10.2196/85425 (PMC13434255; doi:10.2196/85425)
Supplement: Supplementary file 1 [file mhealth_v14i1e85425_app3.pdf]

ARE YOU STRUGGLING WITH

LONG COVID?

Participate in the **Long COVID App & Wearable Study**

WHAT WILL I DO?

Co-design study app experience with the research team

Gain unique insights from your personal health experiences

Complete daily surveys and tasks on your smart-phone (**<5 min/day**)

WHAT ELSE SHOULD I KNOW?

Participants will use a study-provided **Oura ring**, **Empatica EmbracePlus wristband**, and **Lumia ear device**

Any U.S. resident, aged **18+**, who has experienced Long COVID for at least 3 months may be **eligible** to participate

The full study period will last **5 months**

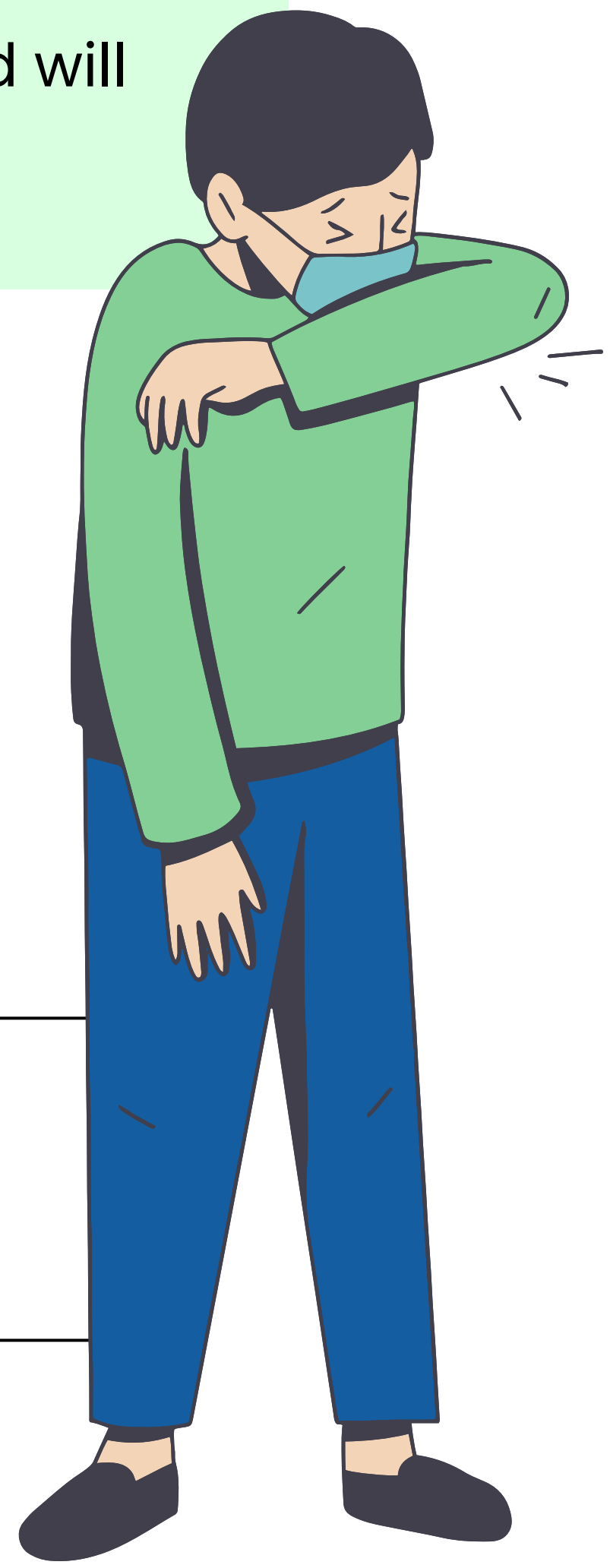

HOW DO I PARTICIPATE?

If you are **interested in participating** in the Long COVID App & Wearable Study, please contact **Emma Karlin** at **emma@4youandme.org** for more information.

*PARTICIPANTS WILL BE COMPENSATED FOR THEIR TIME

WE INVITE YOU TO COLLABORATE ON TESTING AN APP & WEARABLE DEVICES TO BETTER UNDERSTAND THE LONG COVID PATIENT EXPERIENCE

Chan Zuckerberg Initiative

4YOU+ME

PATIENT-LED RESEARCH COLLABORATIVE

ARE YOU STRUGGLING WITH

LONG COVID?

Participate in the **Long COVID App & Wearable Study**

WHAT WILL I DO?

- **Co-design** study app experience with the research team
- **Gain unique insights** from your personal health experiences
- Complete daily surveys and tasks on your smart-phone (**<5 min/day**)

WHAT ELSE SHOULD I KNOW?

- Participants will use a study-provided **Oura ring**, **Empatica EmbracePlus wristband**, and **Lumia ear device**
- Any U.S. resident, aged **18+**, who has experienced Long COVID for at least 3 months may be **eligible** to participate
- The full study period will last **5 months**

HOW DO I PARTICIPATE?

If you are **interested in participating** in the Long COVID App & Wearable Study, please contact **Emma Karlin** at emma@4youandme.org for more information.

*PARTICIPANTS WILL BE COMPENSATED FOR THEIR TIME

WE INVITE YOU TO COLLABORATE ON TESTING AN APP & WEARABLE DEVICES TO BETTER UNDERSTAND THE LONG COVID PATIENT EXPERIENCE

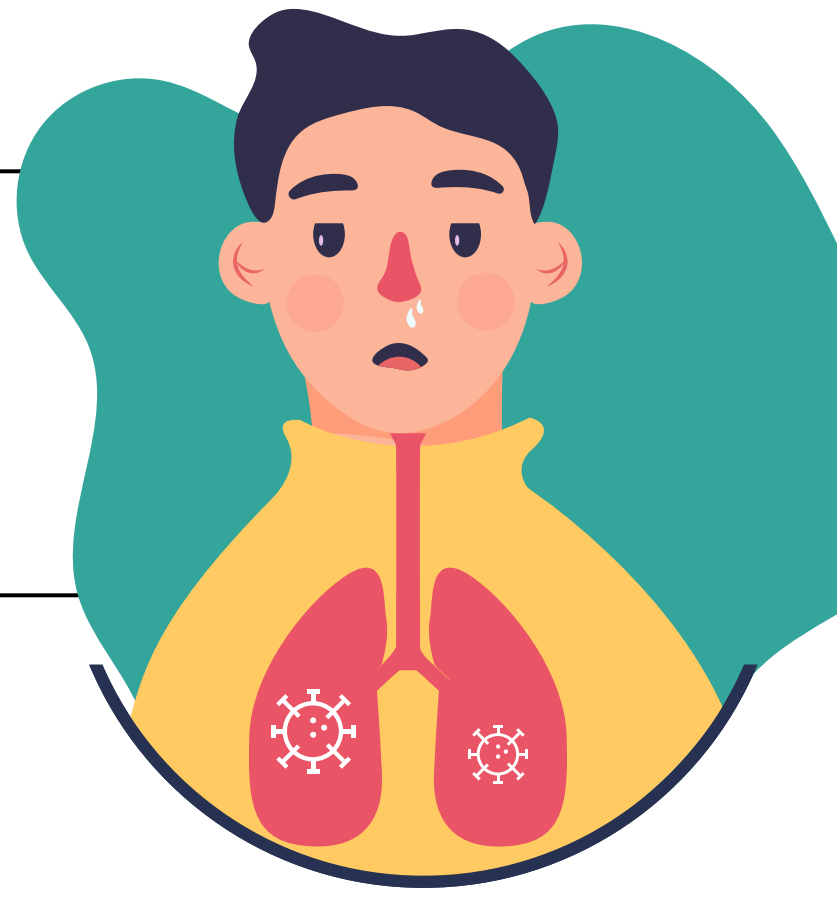

Chan Zuckerberg Initiative

4YOU+ME

PATIENT-LED RESEARCH COLLABORATIVE

ARE YOU STRUGGLING WITH

LONG COVID?

Participate in the **Long COVID App & Wearable Study**

WE INVITE YOU TO COLLABORATE ON TESTING AN APP & WEARABLE DEVICES TO **BETTER UNDERSTAND** THE LONG COVID PATIENT EXPERIENCE

WHAT WILL I DO?

- **Co-design** study app experience with the research team
- **Gain unique insights** from your personal health experiences
- Complete daily surveys and tasks on your smart-phone (**<5 min/day**)

WHAT ELSE SHOULD I KNOW?

- Participants will use a study-provided **Oura ring**, **Empatica EmbracePlus wristband**, and **Lumia ear device**
- Any U.S. resident, aged **18+**, who has experienced Long COVID for at least 3 months may be **eligible** to participate
- The full study period will last **5 months**

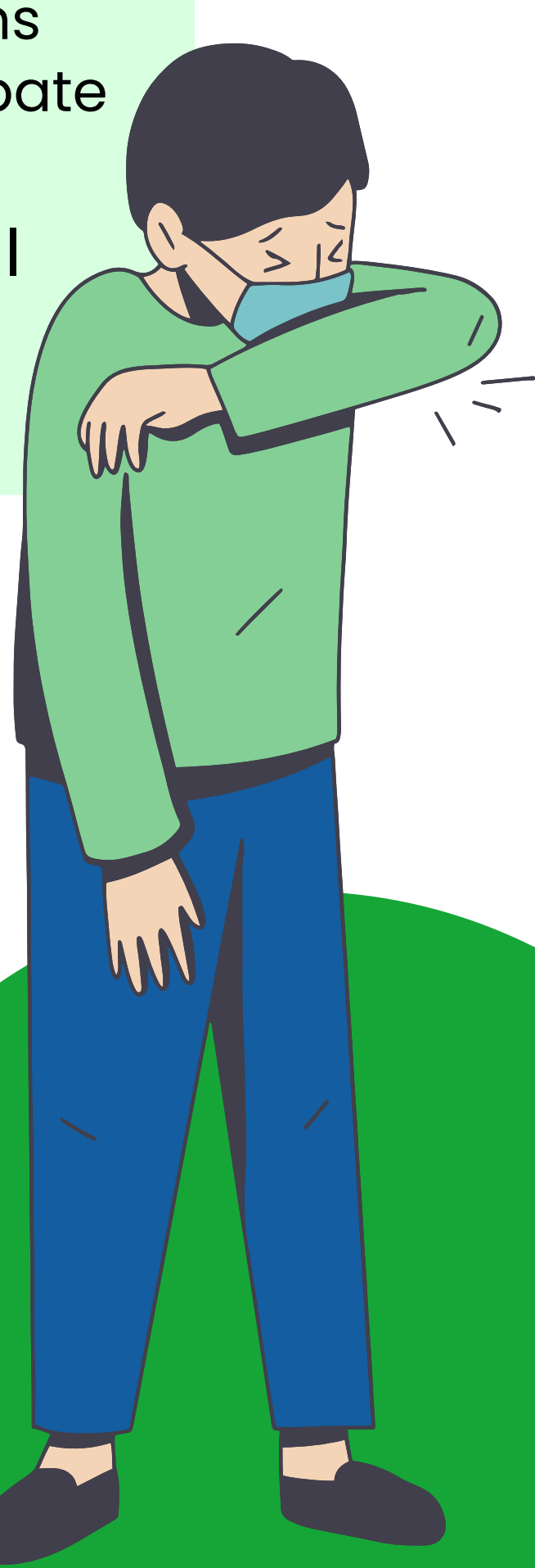

HOW DO I PARTICIPATE?

If you are **interested in participating** in the Long COVID App & Wearable Study, please contact **Emma Karlin** at **emma@4youandme.org** for more information.

*PARTICIPANTS WILL BE COMPENSATED FOR THEIR TIME

Chan Zuckerberg Initiative

4YOU+ME

PATIENT-LED RESEARCH COLLABORATIVE

ARE YOU STRUGGLING WITH

LONG COVID?

Participate in the **Long COVID App & Wearable Study**

WE INVITE YOU TO COLLABORATE ON TESTING AN APP & WEARABLE DEVICES TO BETTER UNDERSTAND THE LONG COVID PATIENT EXPERIENCE

WHAT WILL I DO?

- **Co-design** study app experience with the research team
- **Gain unique insights** from your personal health experiences
- Complete daily surveys and tasks on your smart-phone (**<5 min/day**)

WHAT ELSE SHOULD I KNOW?

- Participants will use a study-provided **Oura ring**, **Empatica EmbracePlus wristband**, and **Lumia ear device**
- Any U.S. resident, aged **18+**, who has experienced Long COVID for at least 3 months may be **eligible** to participate
- The full study period will last **5 months**

HOW DO I PARTICIPATE?

If you are **interested in participating** in the Long COVID App & Wearable Study, please contact **Emma Karlin** at **emma@4youandme.org** for more information.

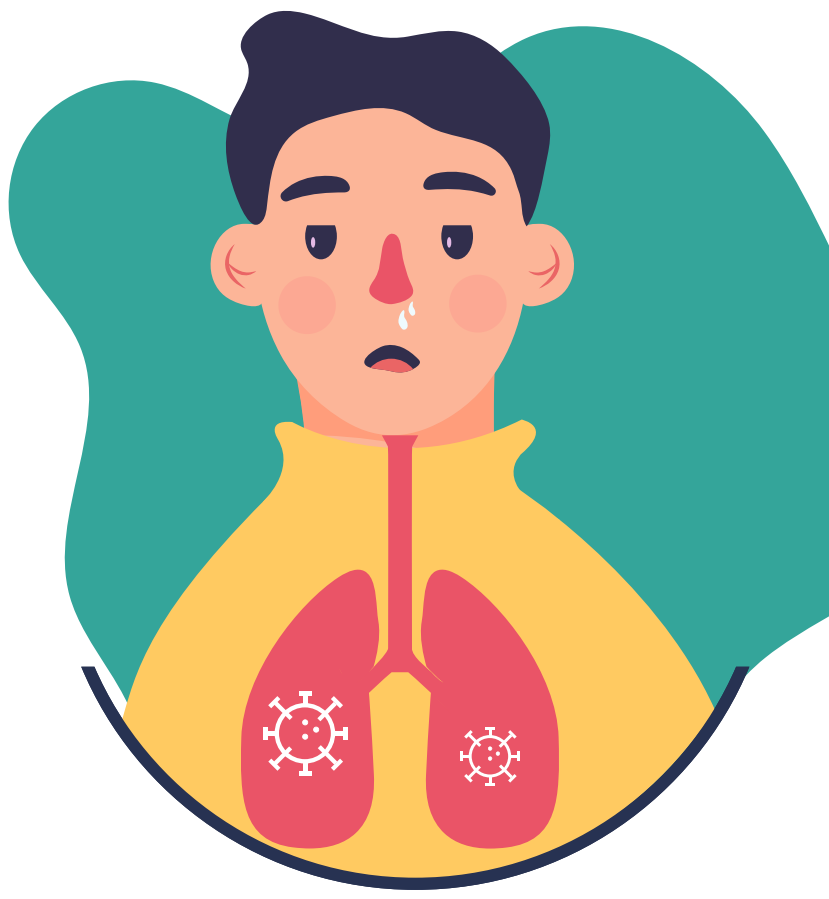

*PARTICIPANTS WILL BE COMPENSATED FOR THEIR TIME

Chan Zuckerberg Initiative

4YOU+ME

PATIENT-LED RESEARCH COLLABORATIVE
