# Supplementary material for: A Personal Health App and Wearable Co-Design Framework for Rare and Complex Diseases: User-Centered, Collaborative Co-Design Study
Source: JMIR Mhealth Uhealth. 2026 Jul 20;14:e85425. doi: 10.2196/85425 (PMC13434255; doi:10.2196/85425)
Supplement: Supplementary file 2 [file mhealth_v14i1e85425_app1.docx]

**1 - PHASE 1 CO-DESIGN ACTIVITIES**

**1A) Module 2: App purpose/function framing independent exercise # 1**

Please indicate on the sliding scale the relative importance of the stated app attribute. If both attributes are of equal importance, select the midpoint.

1a. A tool that aims to detect disease in undiagnosed patients

1b. A tool that aims to track changes in symptoms among diagnosed patients

1a ------------------------------------------------------------------------------1b

2a. A tool that enables the user to track symptoms of their unique disease course

2b. A tool that collects data from users to better understand general symptoms in a population

2a ------------------------------------------------------------------------------2b

3a. A tool that tracks well recognized symptoms longitudinally and remotely

3b. A tool that focuses on tracking poorly understood, reported symptoms longitudinally and remotely

3a ---------------------------------------------------------------------------3b

4a. Focus on testing whether wearable objective data can compliment/replace gold standard outcome assessments

4b. Focus on developing new disease outcome measures from app/wearable data where outcome assessments don’t exist

4a ------------------------------------------------------------------------------4b

5a. A tool that collects data to build a symptom map about a unique disease

5b. A tool that collects data to identify individual level causal links or triggers of symptoms/disease outcomes

5a ------------------------------------------------------------------------------5b

6a. A focus on tracking longitudinal symptoms for unique individuals - patient level symptom maps

6b. A focus on providing alerts when a user’s risk state has changed

6a ------------------------------------------------------------------------------6b

7a. A tool that enables shared insights between patients and clinicians

7b. An app that is intended for end/user (patient) use only

7a ------------------------------------------------------------------------------7

**1B) Module 3 - Defining app attributes independent exercise # 2**

Please complete the following tables

| **App attributes** | **Description** |
| --- | --- |
| **App Themes** |  |
|  |  |
|  |  |
|  |  |
|  |  |
|  | |
| **Primary goal(s) -** What is the primary goal of the app? |  |
| **Target audience(s)** - Who is the end-user of the app? Are there other audiences beyond the end-user that might use the app or associated dashboards? |  |
| **Triggers/context** - What are some potential patient triggers that might instigate or exacerbate symptoms? (E.g., change in diet, viral infection, strenuous activity) |  |
| **Inadvertent consequences** - What are the potential consequences (if any) to the end-user in the use of an app for the intended goal? |  |

| **Symptom List** | **Current measurement tools used** | **Potential existing app/device solutions (used by others)** |
| --- | --- | --- |
|  |  |  |
|  |  |  |
|  |  |  |
|  |  |  |
|  |  |  |
|  |  |  |

**1C) Module 4: Dashboard preferences Independent exercise # 3**

1. *Dashboard Audience:* Please indicate how much the dashboard should be tailored for patients versus doctors.

Patient facing __**_**_%

Doctor facing ____%

2. *Dashboard Symptom Content*: (first drafts): Please review examples provided and provide a first draft sketch or narrative of an example of how your RAO group might want symptom data returned to end-users in order to better co-navigate their health. Please use the following link for further inspiration: <https://echarts.apache.org/examples/en/>
3. Dashboard “Triggers” Content: Consider how you might want to display triggers that modulate the symptoms being followed and whether there are particular insights you wanted returned to the users. Please indicate your thoughts on how triggers should be presented on an app screen.

**1D) Module 5: App specification data dictionary full review independent exercise # 4**

1) Please review the ‘Assessments (Summary)’ and ‘Active Assessments (Item level) tabs of your RAO group’s draft data dictionary and respond with any questions/suggestions. Are there any key triggers/modifiers that you feel are not yet captured by the included assessments? Are you satisfied with the selection of symptom outcome measures? Are there other personal characteristics that are important to track? If there are alternate outcome measures you would prefer to use, please indicate this and why.
2) A medical history survey is included for each RAO group to capture important information that might help characterize patients symptom profiles and experiences. Please provide 4YouandMe with medical history related items that should be included here (if any)
3) Consider how you might want to use “routines”, possible ways to label these routines, and possible ways to label unexpected event

***Please see App specification document template (Supplemental Material X) for an example of what was provided to codesigners to review***

**1E) Module 5: UX preferences independent exercise # 5**

Regarding the I’ve Noticed and Compass log features, we discussed the modalities of video, audio, text, drawing and motion.

1. Which modality/modalities do you think will be most useful for your cohort?
2. Can you suggest any other modalities of expression that would be helpful to have the option of engaging through in the Diary?
3. We plan to have the participant be able to control, entry by entry, whether a diary entry is private/on-the-participant’s-device only versus shared.
4. Is there anything else you’d like to add regarding the Diary feature?

Regarding the Dashboard, we mentioned the option of adding quick tags/notes to your timeline for anything you want to log that seems potentially significant for you/your experience. We will make this as easy and seamless as possible. We anticipate offering the capability of the participant adding real time “now” tags, and also the capability of scrolling back and adding past tags/notes for things that one could not tag in the moment but remembers and wants to tag/note.

5. What tag format/s (examples: a. text, b. audio) do you anticipate you/your cohort will most want to use? Why?
6. Do you anticipate that the entering of “I’ve noticed …” tags will occur mainly (a) in the moment on the phone, (b) on the phone later, (c) on a larger computer screen in the moment, or (d) on a larger computer screen later?

We discussed that in addition to the software suggesting things noticed, we would like participants to be able to choose what to follow. As part of the *“Have you noticed…?”* feature, participants can choose what they want to explore and how. We anticipate it being useful to participants to be able to, for example, set thresholds for chosen metrics and to receive notices when these have been neared, and to be able to look at one’s own dashboard data and try to discover insights.

7. How much do you think the ability to take something you want to work on, turn it into an exploration, and track that exploration will be useful? How useful? To how many?
8. Can you let us know *what* you would like to set up to track?
9. If you are interested in going into more detail, any notes/suggestions on *how* (e.g., in what fashion and with what timing, etc.) to do this would be welcome.

As we discussed in our call, we are hoping you will come up with examples of the type of preparatory exercises you think would be useful in getting folks ready to reflect.

Reflections refer to at will participant entries of daily self reflection – noticing how one is feeling, how one’s day and night have been, et cetera. Participants can choose when in the day/night to do these daily reflections. In support of these, on this slide we have listed some possible optional menu items of preparatory exercises and tool offerings that we could make available for each participant in the app. We offer these options for your consideration as ways to possibly support participants to get into the mood and zone for self reflection before entering their daily reflection.

10. Do you have excitement and/or caution about any options listed? What are your favorite/least favorite of these? Share as much detail as you like.
11. Do you have any other options to suggest that we have not listed? We’d like to know any suggestions that you have for this.
12. Are there any examples from your group of tools people like to use (e.g., video games, Headspace, …) that have been useful mechanisms/exercises for getting into states of being for self reflection and/or connection to oneself?
13. How much do you think folks will want to use the computer screen, versus the phone, to look at their data?
14. If you are interested in going into more detail, any notes/suggestions on how and for what purposes participants may most want to engage with their data is welcome.
