# Supplementary material for: A Personal Health App and Wearable Co-Design Framework for Rare and Complex Diseases: User-Centered, Collaborative Co-Design Study
Source: JMIR Mhealth Uhealth. 2026 Jul 20;14:e85425. doi: 10.2196/85425 (PMC13434255; doi:10.2196/85425)
Supplement: Supplementary file 3 [file mhealth_v14i1e85425_app10.docx]

**12 - App features implemented from direct participant feedback**

| New feature description | Details |
| --- | --- |
| Personalized app notifications | User can choose their own personalized time to receive notifications to complete tasks |
| PCD app MIR spirometer data triggered screen to contact study doctor | Produce an automated trigger when spirometry details surpass a clinically significant value that could indicate risk. Encourage the participant to contact their doctor, or go to the nearest emergency if feeling unwell. |
| Active task skip button | Add a skip button to all in-app cognitive active tasks so participants don't have to view and miss these tasks when they can not safely walk |
| Desktop version of Compass | Compass sometimes is hard to view in the app. Produce a desktop version of the Compass |
| Sharing link for compass | Produce a sharing feature where the user can invite other individuals to view their compass chart |
| V1 - Emoji classification of I've noticed/Reflection entries | Produce ways to label I've noticed and reflection entries for ease. Produce a label feature where the user will be provided with a list of emojis that can be used to label each I've noticed or Reflections entries. |
| V2 - Emoji classification of I've noticed/Reflection entries | The emoji will show up in the compass chart |
| Increase character limit for I've noticed text entries | Increase character limit by 5X for I've noticed text entries |
| Increase the color contrast of the axis and legends in the Compass chart | Change the colour of the axis and labels in the compass chart so there is higher contrast (currently, the green and blue are hard to differentiate) |
| Add new hydration question to quick activities for MyPancreatitis only | Add new hydration questions fo the in-app daily quick activities survey |
| Add Bristol Stool Chart to MyPancreatitis app | Add Bristol Stool chart as a weekly prompted survey |
| Clarify instructions for walk test | Revise the narrative on the first screen of this task to the following "2 Minute Walk Test: This activity monitors your heart rate and measures how far you can walk in 2 minutes. For this task, you must try to walk as fast as you can for 2 minutes, ideally in a straight line. It's easier to perform this task outside so you have more space to walk in a straight line. PLEASE DO NOT ATTEMPT this task if you feel you cannot walk safely unassisted. " |
| Ability to report whether symptoms are disease or non-disease related | Revise daily in-app quick activity survey to prompt the user to indicate whether a symptom is related/caused by their condition: [ ] Select if this symptom is primarily related to or caused by your condition. |
| Add Pain quick activity question to MyLongCOVID and MyPCD apps | Add additional pain question to the in-app daily quick activities survey (MyLongCOVID and MyPCD apps only) |
| Dark mode option | Enable a dark mode option in the app for visually impaired individuals (e.g., LongCOVID) |
| Add screening question to walk/gait tasks | Add a screening question before the gait/walk tests that state: "Can you walk safely unassisted? If no, then the user is given a prompt that states: "We're sorry, we don't think this task is safe for you to complete right now, please try again later when you can walk safely unassisted". then exit out of the task |
| Add screening question to OurVCP pain survey | Do you currently have any pain to report? Yes/no, if no then exit out of survey |
| Add Modified OurVCP pain survey to the MyPancreatitis app | Please add the OurVCP pain survey to the MyPancreatitis app. Can you modify the image to only include the torso (as this is where the pain is localized in pancreatitis)? |
| Update MyPCD Cough survey with new Q about sputum | Add new sputum question to the daily cough survey (MyPCD app only) |
| Update MyLongCOVID app with new quick activities Q about 'wired but tired' | Add a new in-app daily quick activities question: please rate the degree to which you feel "wired but tired" ... |
| Function to see other participant's data | Produce a feature that shows cohort level symptom data and a mechanism for users to consent to this de-identified data sharing |
