# Supplementary material for: A Personal Health App and Wearable Co-Design Framework for Rare and Complex Diseases: User-Centered, Collaborative Co-Design Study
Source: JMIR Mhealth Uhealth. 2026 Jul 20;14:e85425. doi: 10.2196/85425 (PMC13434255; doi:10.2196/85425)
Supplement: Supplementary file 4 [file mhealth_v14i1e85425_app11.docx]

**7 - PROPORTION OF PARTICIPANTS RATING THEIR LIKLIHOO TO USE THE APP IN THE FUTURE ON A 1 (LEAST LIKELY) TO 10 (MOST LIKELY) RATING SCALE**
