# Supplementary material for: A Personal Health App and Wearable Co-Design Framework for Rare and Complex Diseases: User-Centered, Collaborative Co-Design Study
Source: JMIR Mhealth Uhealth. 2026 Jul 20;14:e85425. doi: 10.2196/85425 (PMC13434255; doi:10.2196/85425)
Supplement: Supplementary file 5 [file mhealth_v14i1e85425_app12.docx]

**8 - PARTICIPANT BETA TESTING FEEDBACK THEMES**

| Core Theme | Reflections/Statements | Sub-themes |
| --- | --- | --- |
| **Functionality** | Confusion about app navigation. | App navigation and user interface clarity |
|  | Need clarification on workflows. |  |
|  | Need simpler navigation. |  |
|  | Unclear instructions for walking/gait/spirometry tasks. | Task instructions |
|  | Need for real time prompts and post-task feedback. |  |
|  | Want for access to data (survey results, trends over time). | Data accessibility |
|  | Want for interactive displays (calendars, body maps). |  |
|  | Need for flexibility in how and when they complete surveys (multiple entries per day to only a few times a week, editing past surveys). | Customization |
|  | Want for customizable tracking (tags, labels for activities). |  |
|  | Need for condition-specific tracking (GI issues, pain location, bowel movements, tremors). | Health tracking improvements |
|  | Desire to integrate tracking for medical appointments, dietary logs, hydration, mental health, and actionable insights. |  |
|  | Need for better integration on wearables not in the study, HR monitoring, ability to import data from other apps including nutrition. | Device compatibility |
|  | Better notification system (medication reminders, device disconnection, exertion warnings). | Notifications |
|  | Notifications for those activities that are overdue. |  |
| **Usability** | Confusion around Oura device prompts during enrollment. | Onboarding and instructions clarity |
|  | Requests for clearer, more detailed instructions before and during tasks (gait task, pain questionnaire). |  |
|  | Desire for explanations/definitions within the app (similar to Oura app) to help interpret data and app features. |  |
|  | Need for better color contrast (compass graph colors, legend colors, accommodate color blindness). | Accessibility |
|  | Improve font size and element scale for older users. |  |
|  | Suggestions for dark mode and more modern, dynamic UI elements to keep the app visually engaging. |  |
|  | Include options for emojis or simple images to visually express feelings or symptoms. |  |
|  | Ability to close or skip tasks that users cannot perform (gait and walk tasks), preventing them from being stuck “pending.” | Survey usability |
|  | More flexible response options on surveys (intermediate frequency choices “once in a while,” “a few days”). |  |
|  | Larger pain image map for easier selection. |  |
|  | ‘None’ option for pain therapies instead of just an “other” text box. |  |
|  | Add prompts or clarifications on what timeframe pain questions refer to (current vs. last week). |  |
|  | Request to connect Apple Watch and transfer data into app. | Device compatibility |
|  | Compatibility with iPad, PC, or Mac for enhanced viewing on bigger screens. |  |
|  | Add symptom checklist options rather than free-text in logs to ease user burden. | Symptom tracking |
|  | Requests for symptom tracking over time, including future and current symptoms, with visualization. |  |
|  | Suggestions for new tracked items: diet, weather impact, physical activity, mucus secretion related to foods, continuous HR monitoring. |  |
|  | Show notification badges (red circle with count) clearly on app icon to indicate pending tasks or new messages. | Notifications and reminders |
|  | Suggest a badge or progression system for motivation. |  |
|  | Add calendar integration for questionnaires, tasks, and medical appointments to centralize scheduling. |  |
| **Benefits** | Daily check-ins, mood surveys, and pain tracking increased participants' self-awareness - helping them recognize stress, anxiety, pain, and sleep patterns that they might not otherwise notice. | Self awareness |
|  | Sleep and activity data (especially from Oura) provided clear feedback, allowing participants to notice changes like sleep interruptions |  |
|  | Insights from the study prompted healthier behaviors, such as increased activity, quitting smoking, and being more mindful of routines like sleep or relaxation. | Behavior modification |
|  | Features like the walking task or reminders encouraged physical movement and routine, contributing to improved fitness and daily structure. |  |
|  | Participants found features like quick device charging, simple navigation, and reminders were convenient and easy to use. | Ease of use and study support |
|  | Enjoyment of features (Oura app’s meditation, reminders, and data feedback) and engaging group interactions (Zoom meetings) contributed to ongoing app usage and study satisfaction. |  |
|  | Zoom meetings and study-wide discussions helped clarify study goals, provided a sense of connection, and improved participant understanding of the study’s aims. |  |
|  | Appreciation for covering a wide range of symptoms (mood, strength, activity, pain) in a variety of formats (questionnaires, activity tests, comment boxes, and reflection logs). | Comprehensive symptom tracking |
|  | Real-time feedback (using Oura, Lumia, Empatica, MIR Spirometry) empower users to take charge of their health. |  |
|  | Participants mention feeling more accountable, making healthier choices, becoming more active, and sharing data with their healthcare providers for enhanced care. |  |
| **Harms** | Daily surveys, reflections, and walking/gait tasks felt too frequent, repetitive, and tiring, especially when symptoms didn’t change often. | Burden and fatigue |
|  | Medication tracking was frustrating, being split across many screens and missing key information like dosage and frequency on a single page. |  |
|  | Lack of a dark mode posed issues for people with light sensitivity. | Accessibility and Usability |
|  | Cognitive challenges made it tough to interpret certain app features without clear instructions. |  |
|  | Inability to opt out or adjust task frequency left some users with tasks that didn’t fit their current needs or abilities. | Limited personalization |
|  | Irrelevant or redundant tasks, like frequent walking tests, were frustrating for those at certain illness stages. |  |
|  | Devices sometimes made users feel worse about their health rather than motivated. | Emotional Impact |
|  | Daily reflections could feel emotionally draining or unnecessary. |  |
|  | Persistent, uncompleted tasks reminded users of disease progression. |  |
|  | Incomplete feedback, such as lack of notifications when a gait task ended, led to confusion. | Lack of instruction |
|  | Open-ended logs were difficult for users who need more structure or prompts. |  |
| **Self Explorations** | Participants want frequent, customizable tracking for mood, symptoms, and daily health behaviors (hydration, bowel movements, diet, blood pressure). | Desire for flexible tracking |
|  | Need for reflective prompts, personalized reminders, and easier ways to visualize or export personal data. |  |
|  | Found that multi-point logging and options to add context (notes, voice memos) are highly valued. |  |
|  | Requests the ability to review or export all their logs and answers to spot trends, reflect, and share with care teams. | Self empowerment |
|  | Suggestions include more intuitive visuals (summary snapshots, icons on graphs) and comparisons over time. |  |
|  | Feedback emphasizes tracking various triggers and correlating symptoms (flare-ups, food, hydration, treatments) for deeper insight into health. | Adaptive experience |
|  | Suggestions for tailoring questions by activity, allowing repeats of key survey entries, and incorporating condition-specific prompts. |  |
|  | Participants want access to peer comparisons, support groups, glossaries, and ways to ask questions. | Community support |
