# Supplementary material for: A Personal Health App and Wearable Co-Design Framework for Rare and Complex Diseases: User-Centered, Collaborative Co-Design Study
Source: JMIR Mhealth Uhealth. 2026 Jul 20;14:e85425. doi: 10.2196/85425 (PMC13434255; doi:10.2196/85425)
Supplement: Supplementary file 6 [file mhealth_v14i1e85425_app4.docx]

## **Inclusion criteria**

- 18+ years (except 14+ years for the PCD sub-arm)
- own a personal mobile device and use this phone to download and use the app for the study
- resides in the US
- be willing to use the study wearable devices
- have a clinician confirmed or suspected diagnosis of one of the 5 rare conditions as defined below in the cohort specific inclusion criteria
- In the event of the identification of more than the target sample size for all sub-arms (i.e., n=50), we will prioritize participants who have high disease activity
- *Sub-arm specific inclusion criteria:*
  - **Long COVID sub-arm**
    - have experienced Long COVID for at least 3 months as a continuous, relapsing and remitting, or progressive condition; and
    - have experienced within the past week one or more of the following symptoms: shortness of breath, cough, persistent fatigue, post-exertional malaise (worsening of symptoms or fatigue after minimal activity), difficulty concentrating, memory changes, recurring headache, lightheadedness, fast heart rate, sleep disturbance, problems with taste or smell, bloating, constipation, and diarrhea.
    - symptom(s) of COVID significantly impact your ability to work, ability to give care, enjoy recreational activities or reduce your quality of life
    - Own a personal iPhone 8 (iOS 16+) or Android smartphone (version 12 or newer)
  - **Sarcoidosis sub-arm**
    - have a diagnosis of sarcoidosis confirmed by a doctor, and have experienced fatigue for at least 2 days in the past month
    - Own a personal iPhone 6s (iOS 15+) or Android smartphone (version 12 or newer)
  - **Pancreatitis sub-arm**
    - have a diagnosis of chronic pancreatitis confirmed by a doctor, have had at least 1 pancreatitis flare in the past year, and have not had a surgery that completely removed the pancreas
    - Own a personal iPhone 6s (iOS 15+) or Android smartphone (version 12 or newer)
  - **VCP disease sub-arm**
    - have a clinician confirmed pathogenic VCP mutation or a family history (or suspected family history) of VCP disease, and are experiencing symptoms likely to be associated with the disease
    - Own a personal iPhone 8 (iOS 16+) or Android smartphone (version 12 or newer)
  - **PCD sub-arm**
    - a genetic test that confirms two abnormal copies (one on each gene) of a gene associated with PCD. These abnormalities must be classified as "pathogenic" or "likely pathogenic."; Or an abnormal electron microscopy cilia biopsy done in a PCD center; and symptoms consistent with PCD
    - Own a personal iPhone 6s (iOS 15+) or Android smartphone (version 12 or newer)

**Exclusion criteria**

- Unable to read or understand English
- Too unwell or unable to complete the study activities
