# Supplementary material for: A Personal Health App and Wearable Co-Design Framework for Rare and Complex Diseases: User-Centered, Collaborative Co-Design Study
Source: JMIR Mhealth Uhealth. 2026 Jul 20;14:e85425. doi: 10.2196/85425 (PMC13434255; doi:10.2196/85425)
Supplement: Supplementary file 7 [file mhealth_v14i1e85425_app5.docx]

**3 - WEARABLE/SMART DEVICES**

**Oura Ring**

The Oura Ring Generation 3 measures heart rate, resting heart rate, heart rate variability (HRV), respiration rate, breathing variance, sleep stages and quality metrics, body temperature variation, duration, intensity, blood oxygen levels (SpO2) and real-time activity tracking including: automatic workout detection, timing of activities and inactivity, sedentary time. The Generation 3 Oura Ring also includes 24/7 heart rate monitoring, workout heart rate tracking, and menstruation tracking.

**Empatica EmbracePlus**

The Empatica EmbracePlus is an FDA-approved device designed for advanced health monitoring, especially in individuals with chronic conditions. The Empatica EmbracePlus measures heart rate, skin conductance/electrodermal activity (EDA), blood oxygen saturation (SpO2), pulse rate, pulse rate variability (PRV), respiratory rate, skin temperature, motion (accelerometer and gyroscope data), activity and rest data to provide real-time insights.

**MIR Spirobank Spirometer**

The MIR Spirobank Spirometer is a portable, handheld device used to monitor lung function, and provides metrics in real-time. The MIR Spirometer measures forced expiratory volume (FEV1), peak expiratory flow (PEF).

**Lumia**

Lumia is an ear device designed for individuals diagnosed with certain chronic illnesses such as POTS, dysautonomia, and Long Covid, among other conditions. Lumia primarily measures changes in cerebral blood flow via the external carotid artery. Lumia also captures heart rate variability (HRV), and calculates orthostatic stress via motion, accelerometer and pressure sensor data.
