# Supplementary material for: A Personal Health App and Wearable Co-Design Framework for Rare and Complex Diseases: User-Centered, Collaborative Co-Design Study
Source: JMIR Mhealth Uhealth. 2026 Jul 20;14:e85425. doi: 10.2196/85425 (PMC13434255; doi:10.2196/85425)
Supplement: Supplementary file 8 [file mhealth_v14i1e85425_app6.docx]

**4 - APP GOALS/SPECIFICATIONS**

**4A - App Goals by PDG**

| Prototype App | Goals |
| --- | --- |
| OurVCP | 1) To measure deterioration (progression) of patients with multisystem proteinopathy in order to prepare for clinical trials, AND  2) To better understand fatigue, pain and activity level among patients with multisystem disorders in order to improve the patient's quality of life |
| MyPCD | 1) Validate individual patient cough patterns  2) Characterize sleep patterns among individual patients  3) Determine ways to detect quality of voice  4) Determine whether patterns in cough and sleep lend insight into common phenotypes in the larger patient community |
| MyPancreatitis | 1) Build a tool that can track symptoms between clinic visits so patients can more accurately understand and report symptoms to clinicians  2) Identify triggers of disease  3) Develop baselines and endpoints for RCTs |
| MySarcoidosis | 1) Improve communication between clinician and patient surrounding lived experience of condition  2) Help improve ways to capture flares |
| MyLongCOVID | 1) Develop measures to inform clinical trial outcomes  2) To provide patients with insights into their health that they can choose to share with their clinicians and researchers  3) Provide clinicians with information to assess patient progress remotely |

**4B - App Specifications by PDG**

|  | **OurVCP** | **MyPCD** | **MyPancreatitis** | **MySarcoidosis** | **MyLongCOVID** |
| --- | --- | --- | --- | --- | --- |
| **App Themes** | | | | | |
| Ability to build longitudinal symptom maps | x | x | x | x | x |
| Ability to share symptoms with clinicians | x | x | x | x | x |
| Ability to inform new disease measures | x | x | x | x |  |
| Ability to identify triggers of symptoms/disease |  | x | x | x | x |
| Track poorly understood symptoms | x |  |  | x | x |
| Track well recognized symptoms within and between patients |  | x | x |  | x |
| Ability to validate existing outcome measures |  | x |  | x |  |
| Ability to collect baseline data for RCTs |  |  | x |  |  |
| Ability to alert a user of an impending health event |  |  |  |  | x |
| **Key symptoms to track** | | | | | |
| Fatigue | x | x | x | x | x |
| Cognition | x |  |  | x | x |
| Pain | x |  | x | x |  |
| Activity | x |  |  |  |  |
| Deterioration/Mobility | x |  |  |  |  |
| Respiratory function | x | x |  |  |  |
| Cough |  | x |  |  |  |
| Nasal voice | x | x |  |  |  |
| Sleep | x | x | x | x | x |
| Oxygenation |  | x |  |  |  |
| Inability to eat |  |  | x |  |  |
| Post exertional malaise |  |  |  |  | x |
| Dysautanomia |  |  |  |  | x |
| Quality of life | x | x | x | x | x |
| Depression | x | x | x | x | x |
| Anxiety | x | x | x | x | x |
| Routines | x | x | x | x | x |
| Symptom Modifiers | x | x | x | x | x |
| **Wearable/smart devices** | | | | | |
| Oura ring | x | x | x | x | x |
| Empatica EmbracePlus |  |  | x |  | x |
| MIR Spirobank Spirometer | x | x |  |  |  |
| Lumia Ear device |  |  |  |  | x |
