# Supplementary material for: A Personal Health App and Wearable Co-Design Framework for Rare and Complex Diseases: User-Centered, Collaborative Co-Design Study
Source: JMIR Mhealth Uhealth. 2026 Jul 20;14:e85425. doi: 10.2196/85425 (PMC13434255; doi:10.2196/85425)
Supplement: Supplementary file 9 [file mhealth_v14i1e85425_app7.docx]

**5 - PROTOTYPE APP FEATURES**

**5A) I’ve noticed … feature**

**5B) Compass Log feature**

**5C) Reflections feature**
